# Supplementary material for: Identification and functional analysis of non-coding regulatory small RNA FenSr3 in Bacillus amyloliquefaciens LPB-18
Source: PeerJ. 2023 May 15;11:e15236. doi: 10.7717/peerj.15236 (PMC10194069; doi:10.7717/peerj.15236)
Supplement: Supplemental Information 4 [file peerj-11-15236-s004.zip › KO/CK-vs-T1_map/map04015.html]

KEGG PATHWAY: Rap1 signaling pathway - Reference pathway


|  |  |
| --- | --- |
| **Rap1 signaling pathway - Reference pathway** |  |

[
Pathway menu
| Organism menu
| Pathway entry
| Show description
| User data mapping
]

Rap1 is a small GTPase that controls diverse processes, such as cell adhesion, cell-cell junction formation and cell polarity. Like all G proteins, Rap1 cycles between an inactive GDP-bound and an active GTP-bound conformation. A variety of extracellular signals control this cycle through the regulation of several unique guanine nucleotide exchange factors (GEFs) and GTPase activating proteins (GAPs). Rap1 plays a dominant role in the control of cell-cell and cell-matrix interactions by regulating the function of integrins and other adhesion molecules in various cell types. Rap1 also regulates MAP kinase (MAPK) activity in a manner highly dependent on the context of cell types.

|  |  |  |
| --- | --- | --- |
| Reference pathway | 184% 150% 122% 100% 82% 67% 55% | 图片下载 |
